# Dietary Restriction Status for Hen’s Egg, Cow’s Milk, and Wheat in Relation to Plasma Amino Acid Profiles, Growth, and Insulin-like Growth Factor I in Children with Food Allergy

**DOI:** 10.3390/nu18213712

**Published:** 2026-11-09

**Authors:** Seiya Masukane, Suguru Fujita, Ayano Ueda, Fumihiko Hakuno, Koichi Ito, Yuki Kawashima-Sonoyama, Takeshi Taketani

**Affiliations:** 1 Department of Pediatrics, Shimane University Faculty of Medicine, Izumo 693-8501, Japan; masukane@med.shimane-u.ac.jp (S.M.); ttaketani@med.shimane-u.ac.jp (T.T.); 2 Department of Animal Resource Sciences, Graduate School of Agricultural and Life Sciences, The University of Tokyo, Tokyo 113-8657, Japan; fujita-suguru045@g.ecc.u-tokyo.ac.jp (S.F.); uedaayano@g.ecc.u-tokyo.ac.jp (A.U.); hakuno@g.ecc.u-tokyo.ac.jp (F.H.); 3 Department of Physiological Function of Aging, Graduate School of Agricultural and Life Sciences, The University of Tokyo, Tokyo 113-8657, Japan; akoito@g.ecc.u-tokyo.ac.jp

**Keywords:** amino acid profile, children, cow’s milk avoidance, food allergy, growth, insulin-like growth factor I

## Abstract

**Background/Objectives**: Food avoidance may compromise nutrient intake and growth in children with food allergy; however, its association with plasma amino acid profiles remains poorly understood. We aimed to examine dietary restriction status for hen’s egg, cow’s milk, and wheat in relation to plasma amino acids, growth, and insulin-like growth factor I. **Methods**: This single-center cross-sectional study included 80 children aged < 10 years. Dietary restriction status, defined based on the amount of each food medically permitted for routine daily consumption, was dichotomized as complete avoidance/minimal intake or partial/unrestricted intake. Plasma amino acids were available for 79 participants. Multivariable models included all three foods, with Benjamini–Hochberg correction. **Results**: No broad reduction in plasma amino acid concentrations was evident across dietary restriction categories, although several food-specific associations were observed. Less restrictive cow’s milk status was nominally associated with higher height standard deviation score (SDS) and lower aspartic acid concentration. Less restrictive wheat status was associated with lower weight SDS after multiple-testing correction (*q* = 0.046), although the complete avoidance/minimal intake group comprised only three participants. Among children with a cow’s milk allergy, threonine concentration was 34.2% lower in the partial/unrestricted group than in the complete avoidance/minimal intake group (*q* = 0.002). The minimum essential amino acid z-score was inversely associated with weight SDS and body mass index SDS (*q* = 0.023 for both). No association with the overall 20-amino-acid profile remained significant after multiple-testing correction. **Conclusions**: Dietary restriction status was not accompanied by a broad or uniform reduction in circulating amino acids. Instead, the observed pattern suggested selective rather than generalized differences, warranting confirmation in longitudinal studies with quantitative dietary assessments.

## 1. Introduction

In managing food allergy, determining the amount of the food responsible for the allergic reaction that can be safely consumed based on oral food challenges and other clinical information and preventing unnecessary complete avoidance is crucial. Similarly, the Japanese guidelines for food allergy recommend stepwise oral food challenges and continued intake of the causative food in tolerated forms and amounts [1].

Conversely, avoidance of causative foods can substantially reduce the intake of nutrients normally supplied by these foods, particularly when appropriate substitutes are not provided. Therefore, poorly managed elimination diets may lead to an inadequate intake of energy, proteins, vitamins, and minerals and impaired growth [2]. Lower weight and height z-scores have been reported in children who avoid cow’s milk or multiple foods [3,4,5]. However, normal growth may be maintained when dietary counseling is provided by a dietitian and appropriate substitute foods or nutritional supplements are used [6,7]. Thus, the nutritional impact of an elimination diet is likely to depend on the type and number of foods avoided and on the actual dietary intake and nutritional adequacy of the substitute diet.

Dietary proteins and amino acids contribute to growth as structural components of body tissues and through their involvement in metabolic and endocrine processes. In undernourished children, low height-for-age is associated with lower circulating concentrations of all essential amino acids (EAAs), as well as of arginine, glycine, and glutamine [8]. Among young children in Ethiopia, higher intakes of protein, tryptophan, and energy were associated with higher height-for-age z-scores, whereas serum concentrations of insulin-like growth factor I (IGF-I), a nutrition-sensitive endocrine mediator of growth, were associated with linear growth [9]. A higher intake of milk-derived proteins is also associated with higher IGF-I concentrations in children [10]. Experimental studies in animals and cells have further reported that restriction of a single EAA or EAA deficiency can reduce serum IGF-I concentrations [11,12].

In addition to serving as substrates for protein synthesis, amino acids also act as nutrient signals. Nutrient- and amino acid-sensing pathways and insulin or IGF-I signaling converge on the mechanistic target of rapamycin complex 1 (mTORC1), which regulates protein synthesis, autophagy, cellular metabolism, and cell growth [13,14]. Circulating amino acid patterns may reflect the integrated effects of dietary intake, endogenous metabolism, protein turnover, and tissue utilization. Accordingly, metabolomic and amino acid profiling may complement conventional dietary, anthropometric, and biochemical assessments of nutritional and metabolic status, although they do not directly quantify habitual dietary intake [15,16,17].

Previous studies on pediatric food allergy have primarily been focused on the association between food avoidance and nutrient intake, growth, and micronutrient status. In contrast, the association between dietary restriction status for major allergenic foods and circulating amino acid profiles remains poorly characterized. To the best of our knowledge, this study is among the first to comprehensively report the association between dietary restriction status for hen’s egg, cow’s milk, and wheat and both individual plasma amino acid concentrations and the overall plasma amino acid profile in children with food allergy. Additionally, we evaluated the associations of dietary restriction status with growth measures and the IGF-I standard deviation score (IGF-I SDS) to assess anthropometric and endocrine aspects of growth.

## 2. Materials and Methods

### 2.1. Study Design and Setting

This single-center observational study was conducted in the Department of Pediatrics at Shimane University Hospital. Participants were prospectively enrolled between July 2024 and March 2026 and underwent a cross-sectional assessment. Blood samples were collected at enrollment, when dietary restriction status was assessed.

### 2.2. Participants

Children aged < 10 years with food allergy who attended Shimane University Hospital were screened for eligibility. Patients scheduled to undergo blood sampling as part of their routine clinical care were considered for enrollment. Written informed consent was obtained from parents or legal guardians before enrollment. This study was also explained to each child in an age-appropriate manner according to their level of understanding, and assent was obtained when appropriate.

The exclusion criteria were known chromosomal abnormalities; inborn errors of metabolism; hepatic, renal, or heart failure; use of enteral nutritional formulas; and treatment with growth hormones. Children were also excluded if they had an acute infectious, inflammatory, or other illness at the time of blood sampling or if their appetite had not returned to its usual level following a recent acute illness.

### 2.3. Definition of Food Allergy and Dietary Restriction

Hen’s egg, cow’s milk, and wheat allergies were diagnosed separately by a pediatric allergist based on food-specific clinical information, including a clear history of immediate-type allergic reactions, allergen-specific immunoglobulin E test results, and oral food challenge findings. For each diagnosed food allergy, at least two of these three sources of diagnostic evidence were documented.

Dietary restriction status for hen’s egg, cow’s milk, and wheat at the time of blood sampling was assessed through interviews with parents or guardians and a review of medical records. For children with complete avoidance or minimal intake, the approximate age at restriction initiation and its continuation until blood sampling were retrospectively reviewed. The estimated interval was calculated as the age at blood sampling minus the age at restriction initiation, in months. Dietary restriction status was defined based on the amount of each food medically permitted for routine consumption at the time of blood sampling. This measure was intended to reflect the degree of medically guided dietary restriction and did not represent a quantitative assessment of actual habitual food intake or the amount consumed immediately before blood collection.

The dietary restriction status for each food item was classified into three categories, complete avoidance or minimal intake, partial intake, and unrestricted intake, using prespecified operational criteria informed by the amounts used in oral food challenges [1]. These categories were specifically developed for this study and were not based on a previously validated scale. Detailed criteria for each category are provided in Supplementary Table S1.

### 2.4. Clinical Data Collection

At enrollment, clinical data were collected from medical records and interviews with parents or legal guardians. The data collected included age; sex; height; weight; gestational age; birth length; birth weight; medical history; coexisting allergic diseases; current medications; causative foods; dietary restriction status for hen’s egg, cow’s milk, and wheat; and fasting duration before blood sampling.

Height and weight were measured during the study visit, and body mass index (BMI) was calculated as weight in kilograms divided by height in meters squared. The SDSs for height, weight, and BMI were calculated using age- and sex-specific Japanese reference data from the Body Proportion Calculation File version 3.3 (taikakushisu_v3.3.xlsx, 7 May 2018), developed by the Growth Chart Committee of the Japanese Society for Pediatric Endocrinology and the Committee for Growth Research of the Japanese Association for Human Auxology [18,19].

### 2.5. Blood Sampling and Laboratory Measurements

Plasma and serum were separated from the blood samples. The plasma samples were stored frozen until shipment to the Graduate School of Agricultural and Life Sciences, University of Tokyo. For preparing plasma metabolites, 50 µL of plasma was mixed with 120 µL of methanol containing internal control substances (25 µM of 2-morpholino-ethanesulfonic acid and 100 µM of methionine sulfone) on ice. After centrifugation (16,000× *g* for 10 min at 4 °C), 130 µL of the supernatant was mixed with 250 µL of ultrapure water and subjected to ultrafiltration using 3 kDa cutoff filters (Amicon Ultra 3 K device, Merck, Darmstadt, Germany), followed by evaporation for 30 min and lyophilization for 6 h. The lyophilized specimens were resuspended in ultrapure water and subjected to LC-MS/MS (LCMS-8030, Shimadzu, Kyoto, Japan) using the Method Package for Primary Metabolites ver. 2 (Shimadzu), following the protocol provided by the manufacturer. External calibration curves were generated using standard solutions containing known concentrations of the 20 target amino acids. Plasma amino acid concentrations were calculated from the corresponding calibration curves, with correction for the sample dilution factor.

The amino acids analyzed were histidine, isoleucine, leucine, lysine, methionine, phenylalanine, threonine, tryptophan, valine, arginine, cystine, glutamine, glycine, proline, tyrosine, alanine, asparagine, aspartic acid, glutamic acid, and serine. Cysteine was measured as cystine in its oxidized form.

Serum IGF-I concentrations were measured using an electrochemiluminescence immunoassay at SRL, Inc. (Tokyo, Japan) and are expressed in ng/mL. IGF-I SDSs were calculated from age- and sex-specific Japanese reference data using the Body Proportion Calculation File version 3.3 (taikakushisu_v3.3.xlsx, 7 May 2018), as described in Section 2.4 [20].

### 2.6. Outcomes

The primary outcome was the association of dietary restriction status for hen’s egg, cow’s milk, and wheat with plasma concentrations of 20 amino acids. The secondary outcomes were the associations between dietary restriction status for each food and height SDS, weight SDS, BMI SDS, and IGF-I SDS and the associations between individual plasma amino acid concentrations and these growth-related measures.

### 2.7. Statistical Analyses

Continuous variables are presented as means ± standard deviations or medians (interquartile ranges), as appropriate, and categorical variables as numbers (%). Missing data were not imputed, and complete-case analyses were performed.

Plasma amino acid concentrations were natural log-transformed. In the primary analysis, dietary restriction status for hen’s egg, cow’s milk, and wheat was dichotomized as complete avoidance/minimal intake = 0 and partial/unrestricted intake = 1. Associations with individual amino acid concentrations were evaluated using multivariable linear regression models, including all three foods simultaneously and adjusting for age, sex, BMI SDS, and fasting duration. Regression coefficients were back-transformed as 100 × (exp[β] − 1) and are presented as adjusted percentage differences.

Associations between dietary restriction status for each food and height, weight, BMI, and IGF-I SDS were evaluated in separate multivariable models that included all three foods. The models for height, weight, and BMI SDS were adjusted for age and sex, whereas the IGF-I SDS model was additionally adjusted for fasting duration. Associations between individual amino acids and these outcomes were evaluated after log transformation and standardization of amino acid concentrations. These models were adjusted for age, sex, and fasting duration, with additional adjustments for BMI SDS in the IGF-I SDS model.

Sensitivity analyses omitted the wheat dietary restriction status from the primary model or treated the dietary restriction category for each food as an ordinal variable. Exploratory food-specific analyses were restricted to participants allergic to the corresponding food and were adjusted as in the primary analysis.

As complementary exploratory analyses, the natural log-transformed concentrations of the nine EAAs (histidine, isoleucine, leucine, lysine, methionine, phenylalanine, threonine, tryptophan, and valine) were standardized for each amino acid across participants with complete amino acid profiles. Let *z_ij_* denote the standardized log concentration of EAA *j* for participant *i*. The EAA composite score for participant *i* was defined as $\left(1/9\right)\sum_{j=1}^{9}z_{\mathrm{ij}}$, and the minimum EAA z-score was defined as $\underset{j=1,\ldots ,9}{\min}z_{\mathrm{ij}}$. Thus, for each participant, the minimum EAA z-score represented the lowest standardized concentration among the nine EAAs relative to the study-sample distribution of each amino acid. Associations of these two scores with growth-related outcomes were evaluated using the same covariate structures as those used in the individual amino acid models. Principal component analysis was performed using the standardized log concentrations of all 20 amino acids. Associations with the overall amino acid profile were evaluated using permutational multivariate analysis of variance (PERMANOVA) with Euclidean distances and 4999 permutations, with marginal tests implemented using the adonis2 function in the vegan package. Models of dietary restriction status included all three foods and were adjusted for age, sex, BMI SDS, and fasting duration. Growth-related models used the same covariate structures as the corresponding individual amino acid models. Permutational multivariate analysis of dispersion was used to assess the homogeneity of multivariate dispersion between the dietary restriction groups.

HC3 heteroskedasticity-consistent standard errors were used for the linear regression analyses. *p* values were adjusted using the Benjamini–Hochberg method for each analysis family [21]. For analyses of dietary restriction status, multiple-testing correction was performed separately for each food across the outcomes evaluated within that analysis. Two-sided *p* values < 0.05 were considered nominally significant, and interpretation emphasized false discovery rate-adjusted *q* values. Analyses were performed using EZR, a graphical user interface for R, with R version 4.3.1 [22].

### 2.8. Ethical Considerations

This study was conducted in accordance with the Declaration of Helsinki and the Ethical Guidelines for Life Science and Medical Research Involving Human Subjects in Japan. The study protocol was approved by the Medical Research Ethics Committee of Shimane University Faculty of Medicine (approval no. KS20240521-1; approval date: 12 June 2024). Permission to conduct this study was also obtained from the Dean of the Shimane University Faculty of Medicine before participant enrollment began.

Written informed consent and, when appropriate, written assent were obtained before enrollment, as described above. Study identification numbers were assigned to all biological samples and clinical data, which were separately stored from directly identifying information, including names, patient identification numbers, and dates of birth. The participant identification code list was separately stored from the research data, with access restricted to designated members of the research team.

## 3. Results

### 3.1. Participant Characteristics

Eighty children with food allergies were included in the analysis. The median age was 51.5 months, and 54 (67.5%) participants were male. The median SDS values for height, weight, BMI, and IGF-I were −0.38, −0.18, 0.21, and −0.48, respectively. Participant characteristics stratified by dietary restriction status for each food, including estimated restriction intervals, are presented in Table 1.

The distribution of the three dietary restriction categories for each food is presented in Table 2. Among 53 children with complete avoidance or minimal intake (64 food-specific records), the approximate age at restriction initiation was available for 61 records. The median estimated interval to blood sampling was 44 months (IQR, 17–65 months). Restriction status was maintained until sampling in all 64 records, although six records from five children had estimated intervals of less than six months.

The corresponding distributions restricted to participants with an allergy to the relevant foods are provided in Supplementary Table S2.

The plasma concentrations of all 20 amino acids were available for 79 participants; measurements for one participant were unavailable because of analytical issues. The distributions of plasma amino acid concentrations overall and by dietary restriction status for each food are presented in Table 3, and detailed descriptive statistics are provided in Supplementary Table S3.

### 3.2. Associations Between Dietary Restriction Status and Plasma Amino Acid Concentrations

**Figure 1:**
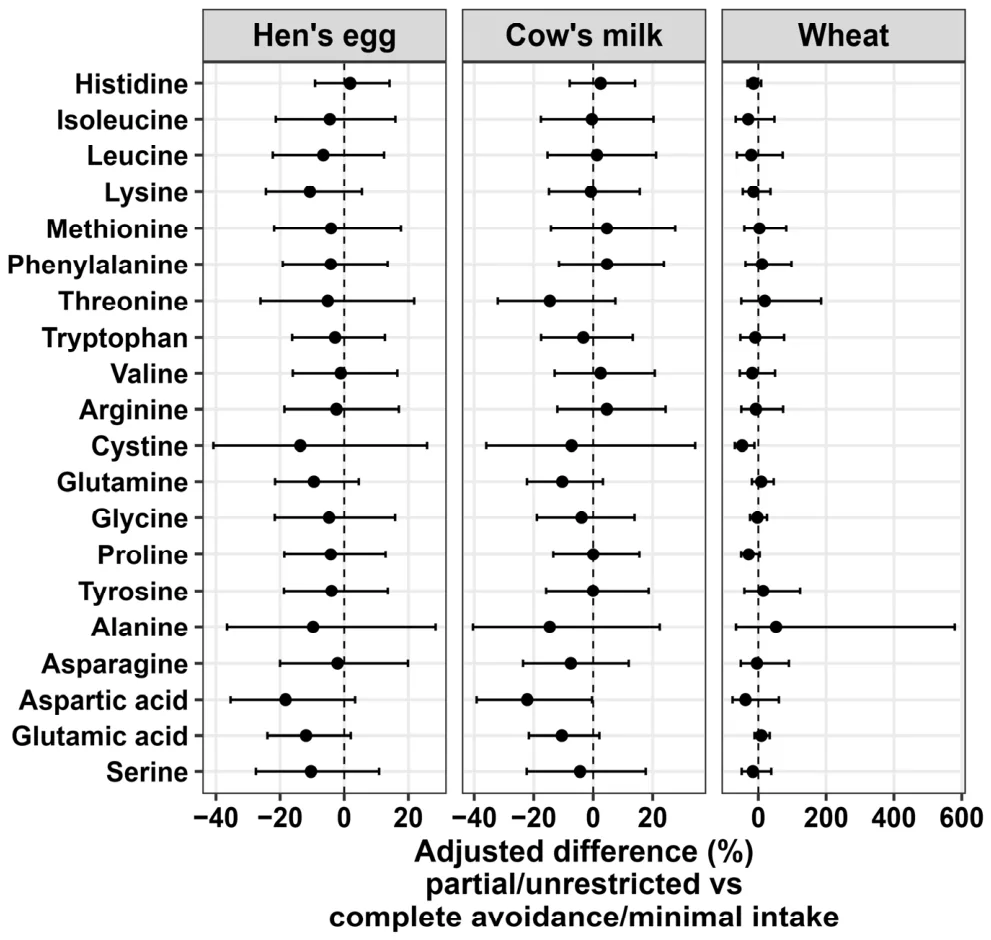
Adjusted associations between dietary restriction status and plasma amino acid concentrations. Dots and horizontal lines indicate adjusted percentage differences and 95% confidence intervals for partial/unrestricted versus complete avoidance/minimal intake. Models included all three foods and were adjusted for age, sex, BMI SDS, and fasting duration. Note that the *x*-axis scales differ among panels. Exact results are provided in Supplementary Table S4. BMI, body mass index; SDS, standard deviation score.

The associations between dietary restriction status and plasma amino acid concentrations are shown in Figure 1 and Supplementary Table S4.

In the primary analysis, no significant associations were found after adjusting for multiple comparisons of any of the three foods. For cow’s milk, the estimated aspartic acid concentration was 22.2% lower in the partial or unrestricted intake group than in the complete avoidance or minimal intake group (95% confidence interval [CI], −39.2% to −0.4%; *p* = 0.046). A nominally lower cystine concentration was also observed in the partial or unrestricted wheat intake group, although only three participants were classified into the complete avoidance or minimal intake group.

### 3.3. Associations Between Dietary Restriction Status and Growth-Related Outcomes

**Figure 2:**
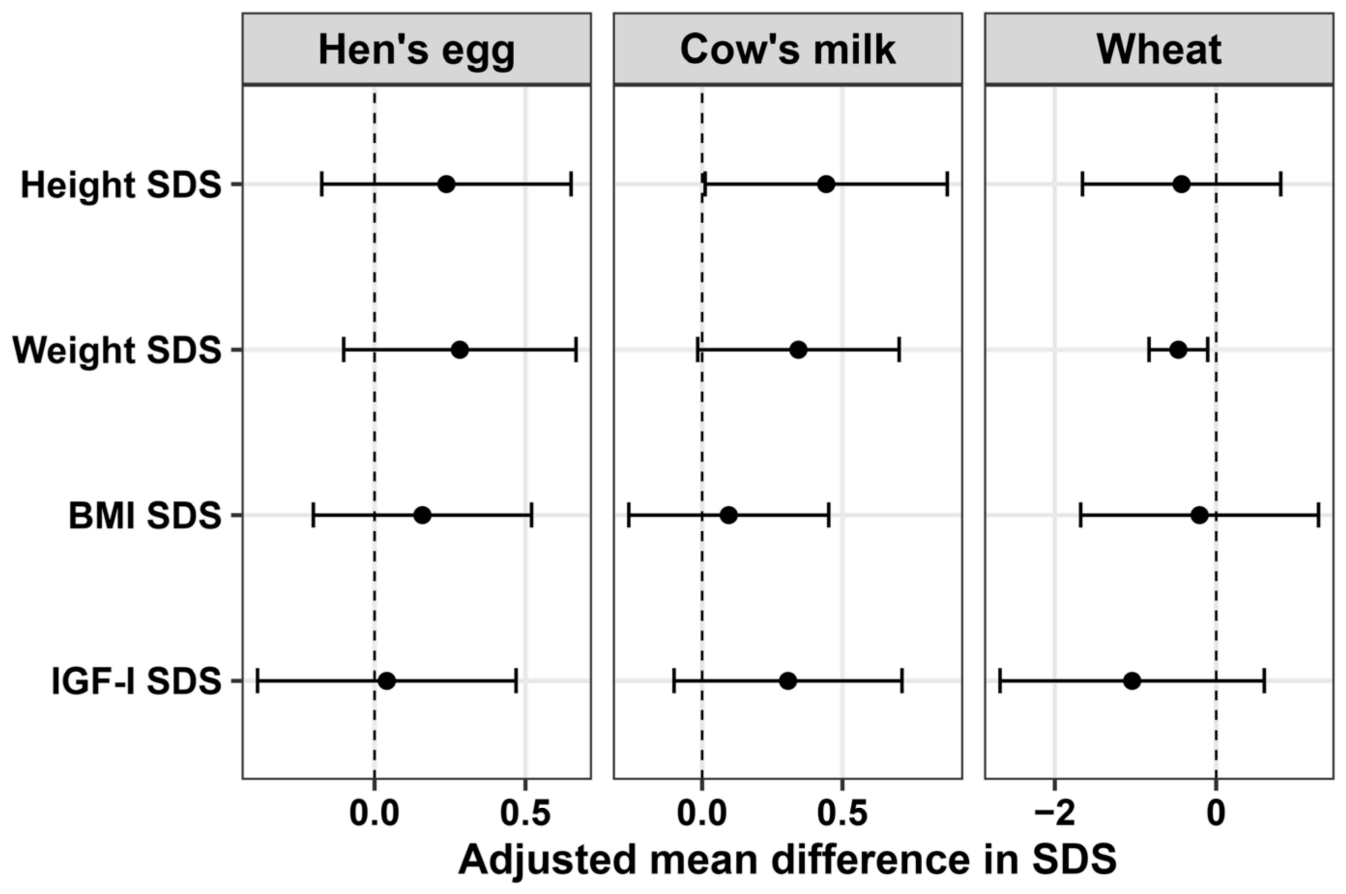
Adjusted associations between dietary restriction status and growth-related outcomes. Dots and horizontal lines indicate adjusted differences and 95% confidence intervals for partial/unrestricted versus complete avoidance/minimal intake. Models included all three foods and were adjusted for age and sex; the IGF-I SDS model was additionally adjusted for fasting duration. Note that the *x*-axis scales differ among panels. Exact results are provided in Supplementary Table S5. BMI, body mass index; IGF-I, insulin-like growth factor I; SDS, standard deviation score.

The associations between the dietary restriction status and growth-related outcomes are shown in Figure 2 and Supplementary Table S5.

For cow’s milk, the height SDS was 0.442 higher among participants classified as having partial or unrestricted intake than among those classified as having complete avoidance or minimal intake (95% CI, 0.011 to 0.874; *p* = 0.045), although this association did not remain significant after multiple-testing correction (*q* = 0.122). For wheat, weight SDS was 0.469 lower among participants classified as having partial or unrestricted intake than among those classified as having complete avoidance or minimal intake (95% CI, −0.832 to −0.105; *p* = 0.012; *q* = 0.046). However, the complete avoidance or minimal intake group included only three participants, and this estimate should therefore be interpreted cautiously. No clear associations were observed for hen’s egg dietary restriction status.

### 3.4. Associations Between Plasma Amino Acid Concentrations and Growth-Related Outcomes

**Figure 3:**
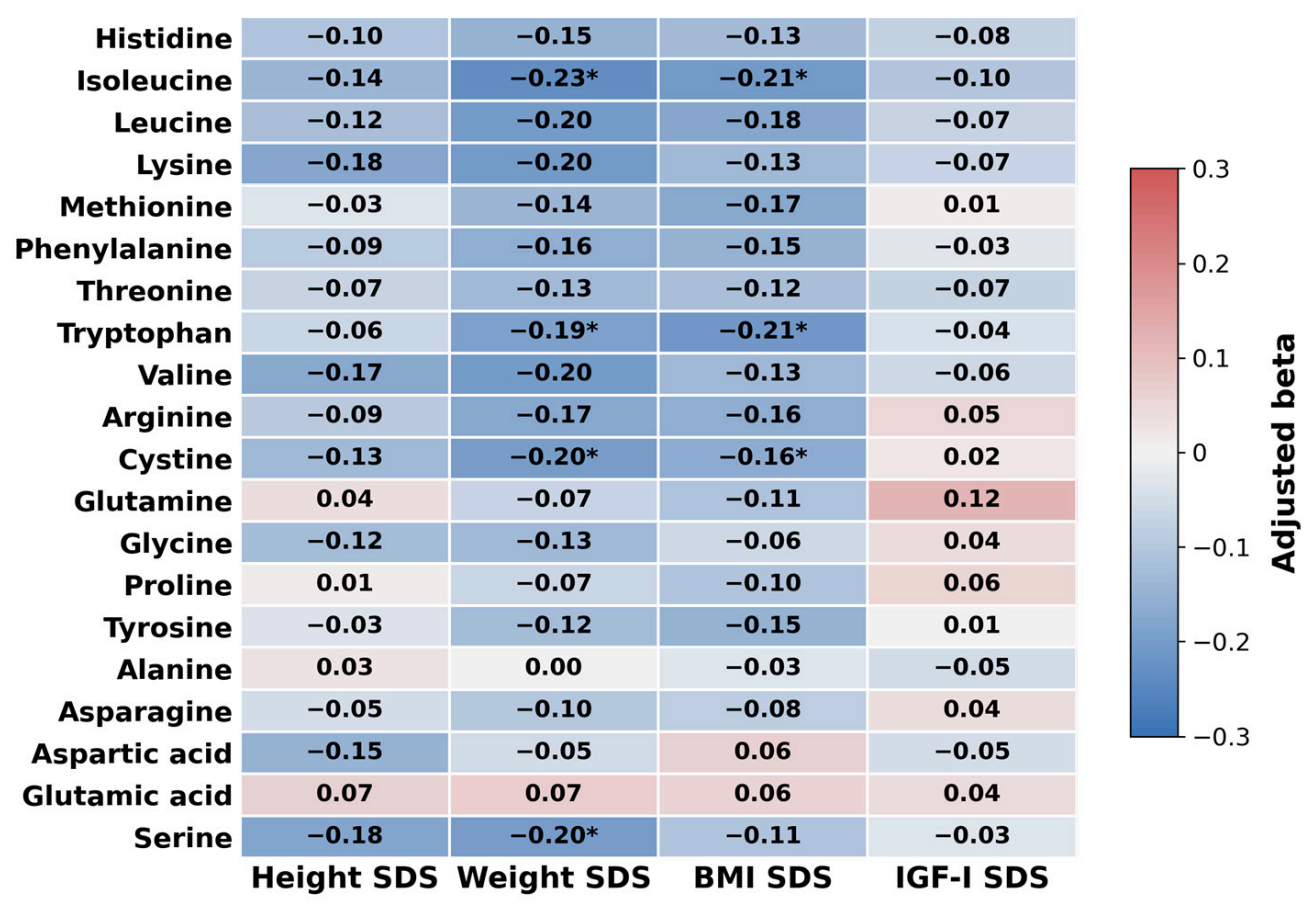
Adjusted associations between plasma amino acid concentrations and growth-related outcomes. Cells show adjusted regression coefficients per 1-SD increase in log-transformed amino acid concentration. Models were adjusted for age, sex, and fasting duration; the IGF-I SDS model was additionally adjusted for BMI SDS. An asterisks (*) indicate nominal *p* < 0.05; no association remained significant after FDR correction. Exact results are provided in Supplementary Table S6. BMI, body mass index; FDR, false discovery rate; IGF-I, insulin-like growth factor I; SDS, standard deviation score.

The associations between the plasma amino acid concentrations and growth-related outcomes are shown in Figure 3 and Supplementary Table S6.

Higher concentrations of isoleucine, tryptophan, cystine, and serine were nominally associated with lower weight SDS and BMI SDS; however, these associations were not significant after adjusting for multiple comparisons. No clear associations were observed between any of the 20 plasma amino acids and height SDS or the IGF-I SDS.

### 3.5. Sensitivity and Exploratory Analyses

In the sensitivity analysis excluding wheat dietary restriction status from the model, the pattern and magnitude of the estimated associations for hen’s egg and cow’s milk dietary restriction status were similar to those in the primary analysis (Supplementary Table S7). In the ordinal sensitivity analysis, each one-category shift toward less restriction for hen’s egg or cow’s milk was associated with a lower aspartic acid concentration. The association for hen’s egg remained significant after adjusting for multiple comparisons, whereas that for cow’s milk did not. A one-category shift toward less wheat restriction was also nominally associated with a lower cystine concentration, although the complete avoidance or minimal intake category included only three participants, and this association was not significant after multiple-testing correction (Supplementary Table S8).

Exploratory analyses were separately performed among participants with a hen’s egg, cow’s milk, or wheat allergy, evaluating dietary restriction status of the corresponding allergenic food in each subgroup. Among children with a cow’s milk allergy, threonine concentration was 34.2% lower among those classified as having partial or unrestricted cow’s milk intake than among those classified as having complete avoidance or minimal intake, and the association remained significant after adjusting for multiple comparisons (95% CI, −46.6% to −18.9%; *p* < 0.001; *q* = 0.002). No significant associations remained after adjusting for multiple comparisons in the analyses of hen’s egg dietary restriction status among children with a hen’s egg allergy or of wheat dietary restriction status among children with a wheat allergy (Supplementary Table S9).

### 3.6. Exploratory Amino Acid Summary and Profile Analyses

The EAA contributing to the minimum EAA z-score varied among participants. Threonine was the most frequent minimum EAA (23/79, 29.1%), followed by lysine (13/79, 16.5%) and phenylalanine (10/79, 12.7%). All nine EAAs were represented as the minimum in at least three participants. The EAA composite score was not clearly associated with height SDS or IGF-I SDS. The minimum EAA z-score was inversely associated with weight SDS and BMI SDS (*q* = 0.023 for both), but not with height SDS or IGF-I SDS (Supplementary Figure S1). The first two principal components explained 63.8% of the variance in the 20-amino-acid profile (Supplementary Figure S2). PERMANOVA showed no significant associations between dietary restriction status, height SDS, or IGF-I SDS and the overall profile. The weight SDS showed a nominal association, which was not significant after multiple-testing correction (partial R^2^ = 0.0318, *p* = 0.040, *q* = 0.130; Supplementary Table S10).

## 4. Discussion

To our knowledge, this study is among the first to report plasma amino acid profiles in relation to dietary restriction status for hen’s egg, cow’s milk, and wheat in children with food allergy. Overall, no broad reduction in plasma amino acid concentrations was evident across dietary restriction categories, although several nominal or exploratory food-specific associations were observed. Several of these findings are related to cow’s milk. Greater restriction of cow’s milk was nominally associated with lower height SDS and higher plasma aspartic acid concentrations; threonine concentrations were also higher with greater restriction among children with a cow’s milk allergy. Taken together, these observations point to differences in selected circulating amino acids associated with food restriction, rather than a simple reduction in circulating amino acids.

Low circulating EAA concentrations are associated with impaired growth in undernourished children [8]. Our study instead included children receiving routine care for food allergy, with restrictions on hen’s egg, cow’s milk, and wheat. Despite these restrictions, the median height, weight, and BMI SDS values were close to zero overall. This distinction is important when interpreting food-specific amino acid findings that may not parallel those reported in generalized undernutrition.

Greater cow’s milk restriction was nominally associated with higher plasma aspartic acid levels, a finding that contradicts the assumption that dietary restriction lowers circulating amino acid concentrations. A similar pattern was observed for hen’s egg in the ordinal sensitivity analysis, in which a shift toward less dietary restriction was associated with lower aspartic acid levels, and the association remained significant after multiple-testing correction. Aspartic acid also behaves differently from other amino acids in undernourished children. Jaffé et al. found higher plasma aspartic acid levels despite reductions in several other amino acids; however, the difference disappeared after nutritional rehabilitation [23]. In severe acute malnutrition, aspartic acid and threonine were the only two of the 17 measured amino acids that were not significantly lower than those in the controls [24]. Taken together, these studies report that aspartic acid need not decline when nutrient availability is limited. Most dietary aspartic acid is metabolized by the intestine before reaching the systemic circulation, whereas circulating aspartic acid is also influenced by endogenous transamination, nitrogen metabolism, the malate–aspartate shuttle, and nucleotide synthesis [25,26,27]. Therefore, the higher aspartic acid concentrations observed with greater restriction may reflect differences in endogenous metabolism or utilization rather than dietary supply alone.

In the exploratory analysis restricted to children with a cow’s milk allergy, plasma threonine concentrations were also higher with greater restriction, and this association remained significant after multiple-testing correction. Threonine is extensively used for intestinal mucin synthesis, and intestinal uptake of circulating threonine increases during mucosal inflammation [28,29]. Altered intestinal barrier function has also been reported in children with food allergy during avoidance of the causative food [30]. These studies link threonine closely to intestinal mucosal metabolism in food allergy but do not explain why its plasma concentration was higher in the more restricted group.

The nominal height difference by cow’s milk dietary restriction status is consistent with previous reports of poorer linear growth in children avoiding cow’s milk, particularly in those with a persistent cow’s milk allergy or multiple food exclusions [3,4,31]. Cow’s milk is a major source of high-quality protein, energy, calcium, and other nutrients required for growth during childhood. Therefore, substantial restrictions place a greater demand on the rest of the diet to provide equivalent nutrition. Together with previous reports, this finding supports continued attention to linear growth in children with substantial cow’s milk restriction. For wheat, less restrictive dietary status was associated with lower weight SDS after FDR correction. However, the complete avoidance/minimal intake group comprised only three participants, resulting in a markedly unbalanced comparison; therefore, this finding should be interpreted cautiously and we refrain from drawing a biological interpretation.

Several amino acids exhibited the same inverse patterns as weight and BMI. Isoleucine, tryptophan, cystine, and serine levels tended to be higher at lower weight SDS or BMI SDS. Notably, the minimum EAA z-score was inversely associated with both weight and BMI SDS after multiple-testing correction. The EAA contributing to this minimum varied among participants, indicating that this association did not reflect a uniformly low concentration of a single specific EAA. Such a pattern is difficult to explain using dietary intake alone. During fasting, circulating amino acids do not change uniformly, as proteolysis and shifts in hepatic and peripheral metabolism can increase or preserve some amino acids, whereas others decline [32]. Heterogeneous amino acid patterns have also been reported in other growth-related settings, including umbilical cord blood studies of fetuses or newborns with impaired fetal growth [33,34]. Therefore, the inverse associations observed here may reflect altered amino acid turnover or utilization accompanied by lower weight and BMI, rather than higher dietary amino acid intake. However, at the profile level, no association survived multiple-testing correction, arguing against a broad shift in the 20-amino-acid profile.

No clear association was observed between IGF-I SDS and either dietary restriction status or plasma amino acid concentrations. Previous pediatric studies have also reported inconsistent associations between milk and protein intakes and circulating IGF-I [10,35,36]. Amino acid sensing and IGF-I signaling converge on mTORC1, but enter this pathway through distinct upstream mechanisms, allowing changes in amino acid availability to occur without parallel changes in circulating IGF-I [13,14]. This may explain why the amino acid and growth associations observed in this study were not mirrored by IGF-I.

The strengths of this study include the evaluation of three major allergenic foods within the same cohort, measurement of 20 plasma amino acids using LC–MS/MS, adjustment for potential confounders, and correction for multiple testing. This study has some limitations. First, the modest sample size and small or unbalanced exposure groups, particularly for wheat dietary restriction status and the food-specific allergy subgroups, may have limited the precision of the estimates. In particular, the complete avoidance/minimal intake group for wheat comprised only three participants; therefore, the association between wheat dietary restriction status and weight SDS, although it remained significant after FDR correction, should be interpreted cautiously because the estimate may be sensitive to individual observations. Accordingly, most findings should be considered hypothesis-generating. Second, this single-center, cross-sectional design precludes causal or temporal inferences, and enrollment during routine clinical blood sampling may have introduced selection bias. The estimated restriction duration was based on approximate initiation ages and did not necessarily reflect the actual duration of non-consumption. Moreover, the timing of food introduction and changes in intake could not be reliably determined in the partial/unrestricted intake groups. Therefore, potential confounding by dietary exposure duration cannot be excluded. Third, dietary restriction status was defined based on medically permitted amounts rather than on quantitatively measured actual habitual intake. Actual consumption may have differed from the medically permitted amount because of individual preferences, caregiver behavior, or other factors. Total energy, protein, amino acid, micronutrient (e.g., calcium, iron, zinc, and vitamins D and B12), substitute food, and nutritional supplement (e.g., calcium and vitamin D) intakes were not assessed. Dietary bioactive compounds, including polyphenols, and blood markers of micronutrient status (e.g., 25-hydroxyvitamin D, ferritin, zinc, and vitamin B12) were also not evaluated. Because dietary restriction status may also reflect food-specific allergy status and severity, residual confounding by these clinical factors cannot be excluded. Potential immunological effects of dietary restriction, including changes in cytokine concentrations or immune-cell profiles, were not assessed. Finally, plasma amino acid and total IGF-I concentrations were measured at a single time point, whereas growth velocity and relevant metabolic or mechanistic measures, including protein turnover, amino acid oxidation, and mTORC1 activity, were not assessed. Prospective studies with quantitative dietary assessments and repeated metabolic and growth measurements are required to confirm these findings.

## 5. Conclusions

In children with food allergy, dietary restriction status was not accompanied by a broad or uniform reduction in plasma amino acid concentrations. Instead, selected food-specific and growth-related associations were observed, suggesting that dietary restriction may be linked to selective rather than generalized differences in circulating amino acids. These findings warrant confirmation in longitudinal studies with quantitative dietary assessments.

## Figures and Tables

**Table 1:** Characteristics of the study participants by dietary restriction status.

Characteristic	Overall, *N* = 80	Hen’s Egg		Cow’s Milk		Wheat	
		Avoidance/ Minimal, *n* = 31	Partial/ Unrestricted, *n* = 49	Avoidance/ Minimal, *n* = 30	Partial/ Unrestricted, *n* = 50	Avoidance/ Minimal, *n* = 3	Partial/ Unrestricted, *n* = 77
Age, months	51.5 (24.0 to 69.0)	57.0 (34.0 to 69.0)	43.0 (17.0 to 66.0)	37.5 (17.2 to 74.0)	55.5 (28.2 to 65.8)	57.0 (36.5 to 77.0)	50.0 (24.0 to 69.0)
Male sex, ***n*** (%)	54 (67.5)	20 (64.5)	34 (69.4)	23 (76.7)	31 (62.0)	2 (66.7)	52 (67.5)
Height SDS	−0.38 (−1.22 to 0.27)	−0.62 (−1.34 to 0.26)	−0.20 (−1.21 to 0.27)	−0.79 (−1.27 to −0.05)	−0.29 (−1.11 to 0.34)	0.02 (−0.55 to 0.55)	−0.39 (−1.23 to 0.27)
Weight SDS	−0.18 (−0.69 to 0.34)	−0.27 (−0.85 to 0.17)	−0.13 (−0.62 to 0.40)	−0.23 (−0.67 to 0.27)	−0.15 (−0.68 to 0.43)	0.39 (0.27 to 0.39)	−0.20 (−0.71 to 0.27)
BMI SDS	0.21 (−0.29 to 0.64)	0.13 (−0.46 to 0.49)	0.31 (−0.23 to 0.73)	0.21 (−0.27 to 0.60)	0.23 (−0.36 to 0.70)	0.46 (−0.17 to 0.99)	0.20 (−0.27 to 0.62)
Fasting duration, min	195 (150 to 255)	210 (180 to 270)	195 (150 to 240)	215 (150 to 281)	195 (162 to 240)	280 (200 to 300)	195 (150 to 250)
IGF-I SDS	−0.48 (−1.08 to 0.17)	−0.35 (−1.22 to 0.19)	−0.49 (−1.03 to 0.13)	−0.52 (−1.22 to −0.06)	−0.44 (−1.05 to 0.20)	0.32 (−0.13 to 1.23)	−0.49 (−1.10 to 0.13)
Hen’s egg allergy, ***n*** (%)	57 (71.3)	31 (100.0)	26 (53.1)	17 (56.7)	40 (80.0)	1 (33.3)	56 (72.7)
Cow’s milk allergy, ***n*** (%)	42 (52.5)	9 (29.0)	33 (67.3)	30 (100.0)	12 (24.0)	1 (33.3)	41 (53.2)
Wheat allergy, ***n*** (%)	14 (17.5)	4 (12.9)	10 (20.4)	7 (23.3)	7 (14.0)	3 (100.0)	11 (14.3)
Estimated time since restriction initiation, months	—	47.5 (26.0 to 58.0)	—	32.0 (11.0 to 68.0)	—	52.0 (29.5 to 69.5)	—

Data are presented as medians (interquartile ranges), unless otherwise indicated. Dietary restriction status was classified separately for each food, and the food-specific groups were not mutually exclusive. The estimated restriction interval was calculated from the approximate age at initiation of medically prescribed restriction to blood sampling. It was available for 28, 30, and 3 records in the hen’s egg, cow’s milk, and wheat complete avoidance/minimal intake groups, respectively. Dashes indicate that comparable restriction intervals were not estimated. Food allergies were not mutually exclusive. BMI, body mass index; IGF-I, insulin-like growth factor I; SDS, standard deviation score.

**Table 2:** Dietary restriction status for hen’s egg, cow’s milk, and wheat.

Food	Complete Avoidance or Minimal Intake	Partial Intake	Unrestricted Intake
Hen’s egg	31 (38.8)	17 (21.3)	32 (40.0)
Cow’s milk	30 (37.5)	5 (6.3)	45 (56.3)
Wheat	3 (3.8)	8 (10.0)	69 (86.3)

Data are presented as ***n*** (%). Dietary restriction status reflects the usual daily amount medically permitted at blood sampling. For the primary analyses, partial and unrestricted intake were combined into one category, as shown in Table 1. Percentages may not total 100% because of rounding.

**Table 3:** Plasma amino acid concentrations by dietary restriction status.

Amino Acid	Overall, *N* = 79	Hen’s Egg		Cow’s Milk		Wheat	
		Avoidance/ Minimal, *n* = 30	Partial/ Unrestricted, *n* = 49	Avoidance/ Minimal, *n* = 30	Partial/ Unrestricted, *n* = 49	Avoidance/ Minimal, *n* = 3	Partial/ Unrestricted, *n* = 76
Histidine	11.66 (9.78–14.14)	11.89 (9.56–14.06)	11.66 (10.10–14.18)	11.43 (9.62–13.92)	12.16 (10.05–14.18)	13.88 (12.60–14.13)	11.64 (9.76–14.11)
Isoleucine	44.85 (31.17–60.24)	45.34 (31.12–60.28)	44.51 (31.59–59.83)	46.20 (31.76–58.67)	44.31 (30.92–61.57)	66.50 (49.89–76.37)	44.68 (31.03–59.35)
Leucine	63.89 (47.99–84.83)	71.18 (49.49–88.29)	62.79 (47.88–77.73)	61.52 (48.90–81.22)	64.55 (46.70–88.58)	90.33 (65.71–99.86)	63.34 (48.05–82.26)
Lysine	14.29 (11.30–17.73)	14.86 (11.47–19.75)	14.16 (11.30–16.29)	13.91 (11.05–17.68)	14.38 (11.52–17.69)	18.52 (14.96–19.14)	14.26 (11.26–17.66)
Methionine	13.70 (10.11–18.83)	13.54 (10.93–19.24)	13.98 (10.09–18.83)	13.57 (10.29–16.65)	13.98 (10.09–19.63)	13.32 (10.62–15.95)	13.84 (10.12–18.99)
Phenylalanine	30.75 (23.28–37.85)	30.34 (23.94–35.65)	30.75 (20.59–38.05)	30.13 (21.08–35.72)	31.68 (26.26–38.05)	27.37 (21.93–30.68)	30.76 (23.55–37.91)
Threonine	44.41 (32.93–53.41)	45.26 (31.34–57.56)	44.41 (33.01–53.39)	46.78 (36.72–62.34)	38.74 (29.89–53.15)	35.83 (32.87–44.94)	44.41 (32.97–53.40)
Tryptophan	14.08 (10.60–17.42)	14.49 (11.61–16.35)	13.80 (10.30–17.63)	14.35 (9.48–17.04)	13.40 (11.38–17.63)	17.63 (13.05–18.00)	14.05 (10.63–17.29)
Valine	135.64 (103.55–167.00)	140.86 (104.35–166.80)	130.49 (103.76–166.65)	129.23 (105.34–168.39)	145.41 (103.33–164.76)	181.62 (141.36–184.96)	133.07 (103.65–166.39)
Arginine	32.94 (23.75–40.36)	35.17 (25.74–43.15)	31.95 (23.68–39.45)	30.17 (23.86–39.05)	35.02 (21.72–41.14)	34.13 (27.05–39.63)	32.73 (23.78–40.27)
Cystine	2.10 (1.39–3.83)	2.31 (1.61–3.95)	1.96 (1.30–3.57)	2.10 (1.50–3.73)	2.10 (1.09–3.82)	3.49 (3.00–4.05)	2.04 (1.34–3.82)
Glutamine	202.33 (181.86–244.04)	212.97 (188.22–277.40)	199.60 (175.04–231.98)	197.24 (181.23–287.04)	203.00 (183.20–233.85)	209.45 (178.28–220.82)	202.16 (182.53–245.79)
Glycine	99.97 (79.33–129.44)	97.67 (77.25–149.34)	100.65 (85.54–118.85)	98.55 (86.20–126.97)	100.65 (72.56–129.20)	104.67 (100.12–110.11)	99.67 (78.72–130.67)
Proline	28.84 (23.09–38.43)	30.81 (23.07–39.56)	27.50 (23.17–36.84)	28.06 (23.14–36.87)	28.84 (22.88–38.65)	43.39 (33.75–49.74)	28.58 (23.01–37.22)
Tyrosine	31.25 (24.95–39.37)	30.44 (25.47–40.17)	31.54 (24.92–39.17)	30.25 (25.84–40.95)	33.00 (24.92–39.15)	28.85 (22.45–32.52)	31.40 (24.97–39.99)
Alanine	57.09 (28.40–84.37)	58.48 (34.14–84.99)	57.09 (27.45–83.53)	69.63 (38.98–85.50)	52.09 (27.59–80.48)	30.53 (27.01–53.94)	57.11 (28.57–85.31)
Asparagine	37.33 (27.29–47.10)	34.74 (27.09–49.42)	37.33 (27.55–46.72)	37.53 (28.70–46.54)	34.43 (26.02–49.50)	37.69 (30.84–40.34)	37.13 (27.30–47.88)
Aspartic acid	7.49 (5.30–10.42)	7.91 (5.80–11.70)	7.48 (4.94–9.53)	8.12 (6.39–12.61)	7.03 (5.00–9.06)	8.41 (7.75–17.31)	7.49 (5.19–10.42)
Glutamic acid	16.99 (14.76–20.41)	18.36 (15.28–22.53)	16.59 (14.40–19.87)	19.42 (15.73–22.38)	16.67 (14.30–19.24)	15.68 (15.52–17.16)	17.09 (14.50–20.48)
Serine	113.77 (87.29–151.03)	114.34 (106.03–155.92)	111.04 (83.38–148.29)	112.40 (94.42–147.34)	114.94 (84.87–155.75)	129.19 (112.11–146.84)	113.77 (84.87–150.35)

IQR, interquartile range. Data are presented as medians (interquartile ranges) in µmol/L. Dietary restriction groups were defined separately for each food and were not mutually exclusive across foods. The partial/unrestricted groups included children without an allergy to the corresponding food. Group sizes reflect participants with available amino acid data.

## Data Availability

The data generated and analyzed in this study are not publicly available because of ethical and privacy restrictions. Any request for data access requires additional review and approval by the relevant ethics committee and participating institution.

## Supplementary Materials

The following supporting information can be downloaded at https://www.mdpi.com/article/10.3390/nu18213712/s1, Supplementary Figure S1: Adjusted associations between essential amino acid summary measures and growth-related outcomes; Supplementary Figure S2: Principal component loading plot of the 20-amino-acid profile; Supplementary Table S1: Operational definitions of dietary restriction status for hen’s egg, cow’s milk, and wheat; Supplementary Table S2: Dietary restriction status of the corresponding food among participants with hen’s egg, cow’s milk, or wheat allergy; Supplementary Table S3: Detailed descriptive statistics for plasma amino acid concentrations; Supplementary Table S4: Adjusted associations between dietary restriction status and plasma amino acid concentrations; Supplementary Table S5: Adjusted associations between dietary restriction status and growth-related outcomes; Supplementary Table S6: Adjusted associations between plasma amino acid concentrations and growth-related outcomes; Supplementary Table S7: Sensitivity analysis excluding wheat dietary restriction status from the model; Supplementary Table S8: Ordinal three-category sensitivity analysis; Supplementary Table S9: Food-specific allergy-restricted analyses; Supplementary Table S10: Multivariate associations with the overall plasma amino acid profile.

## Abbreviations

The following abbreviations are used in this manuscript:

SDS	standard deviation score
EAA	essential amino acid
BMI	body mass index
PERMANOVA	permutational multivariate analysis of variance
