# Supplementary material for: Dietary Restriction Status for Hen’s Egg, Cow’s Milk, and Wheat in Relation to Plasma Amino Acid Profiles, Growth, and Insulin-like Growth Factor I in Children with Food Allergy
Source: Nutrients. 2026 Nov 9;18(21):3712. doi: 10.3390/nu18213712 (PMC13765389; doi:10.3390/nu18213712)
Supplement: Supplementary file 1 [file nutrients-18-03712-s001.zip › nutrients-4639548-supplementary.pdf]

Supplementary Tables

Supplementary Table S1. Operational definitions of dietary restriction status for hen's egg, cow's milk, and wheat.

Food	Complete avoidance or minimal intake	Partial intake	Unrestricted intake
Hen's egg	Complete avoidance, or a medically permitted daily amount of < 1/25 of a heated whole egg or < 1.5 g of heated egg white	A medically permitted daily amount of at least 1/25 of a heated whole egg or 1.5 g of heated egg white, but < 30 g of heated whole egg or 25 g of heated egg white	No medically prescribed upper limit on the amount consumed in daily life
Cow's milk	Complete avoidance, or a medically permitted daily amount of < 3 mL	A medically permitted daily amount of at least 3 mL but < 100 mL	No medically prescribed upper limit on the amount consumed in daily life
Wheat	Complete avoidance, or a medically permitted daily amount of equivalent to < 3 g of boiled udon noodles	A medically permitted daily amount equivalent to at least 3 g but < 100 g of boiled udon noodles	No medically prescribed upper limit on the amount consumed in daily life

Dietary restriction status reflects the usual daily amount medically permitted at blood sampling, rather than intake immediately before blood collection. Unrestricted intake indicates no medically prescribed quantitative upper limit. Categories were defined for this study and were not based on a validated scale.

Supplementary Table S2. Dietary restriction status of the corresponding food among participants with hen's egg, cow's milk, or wheat allergy.

**Food allergy**	*N*	**Complete avoidance or minimal intake, *n* (%)**	**Partial intake, *n* (%)**	**Unrestricted intake, *n* (%)**
Hen's egg allergy	57	31 (54.4)	17 (29.8)	9 (15.8)
Cow's milk allergy	42	30 (71.4)	5 (11.9)	7 (16.7)
Wheat allergy	14	3 (21.4)	8 (57.1)	3 (21.4)

Data are presented as *n* (%) among participants with the corresponding food allergy. Dietary restriction status reflects the usual daily amount medically permitted at blood sampling. Food allergy diagnoses were not mutually exclusive.

Supplementary Table S3. Detailed descriptive statistics for plasma amino acid concentrations.

**Amino acid**	*N*	**Mean ± SD, µmol/L**	**Median (IQR), µmol/L**	**Minimum–maximum, µmol/L**	**Missing**
Histidine	79	12.11 ± 2.96	11.66 (9.78–14.14)	6.55–19.11	1
Isoleucine	79	47.90 ± 20.34	44.85 (31.17–60.24)	18.65–112.47	1
Leucine	79	68.16 ± 27.14	63.89 (47.99–84.83)	27.76–147.04	1
Lysine	79	14.86 ± 4.81	14.29 (11.30–17.73)	7.70–29.89	1
Methionine	79	14.92 ± 6.18	13.70 (10.11–18.83)	5.26–29.23	1
Phenylalanine	79	30.69 ± 10.32	30.75 (23.28–37.85)	12.64–68.02	1
Threonine	79	49.81 ± 28.98	44.41 (32.93–53.41)	15.11–164.69	1
Tryptophan	79	14.24 ± 4.49	14.08 (10.60–17.42)	6.25–25.68	1
Valine	79	140.95 ± 50.30	135.64 (103.55–167.00)	56.55–323.47	1
Arginine	79	32.73 ± 11.07	32.94 (23.75–40.36)	13.64–56.03	1
Cystine	79	2.56 ± 1.77	2.10 (1.39–3.83)	0.36–8.91	1
Glutamine	79	218.82 ± 66.81	202.33 (181.86–244.04)	121.19–484.54	1
Glycine	79	108.99 ± 39.32	99.97 (79.33–129.44)	45.41–257.66	1
Proline	79	31.71 ± 11.00	28.84 (23.09–38.43)	17.35–63.51	1
Tyrosine	79	32.53 ± 10.73	31.25 (24.95–39.37)	12.23–60.37	1
Alanine	79	67.56 ± 50.75	57.09 (28.40–84.37)	10.35–252.80	1
Asparagine	79	37.57 ± 14.09	37.33 (27.29–47.10)	10.35–78.30	1
Aspartic acid	79	8.41 ± 4.33	7.49 (5.30–10.42)	1.83–26.21	1
Glutamic acid	79	18.38 ± 5.68	16.99 (14.76–20.41)	9.97–43.96	1
Serine	79	122.95 ± 51.10	113.77 (87.29–151.03)	43.10–350.39	1

IQR, interquartile range; SD, standard deviation.

Supplementary Table S4. Adjusted associations between dietary restriction status and plasma amino acid concentrations.

Amino acid	*N*	Adjusted difference, % (95% CI)	*P* value	FDR *q* value
**Cow's milk**				
Histidine	79	2.5 (−7.9 to 14.1)	0.652	0.985
Isoleucine	79	−0.4 (−17.6 to 20.3)	0.964	0.998
Leucine	79	1.3 (−15.4 to 21.2)	0.891	0.998
Lysine	79	−0.8 (−14.9 to 15.7)	0.921	0.998
Methionine	79	4.6 (−14.2 to 27.6)	0.655	0.985
Phenylalanine	79	4.6 (−11.5 to 23.8)	0.595	0.985
Threonine	79	−14.6 (−32.1 to 7.5)	0.178	0.892
Tryptophan	79	−3.3 (−17.5 to 13.3)	0.676	0.985
Valine	79	2.5 (−13.0 to 20.7)	0.767	0.998
Arginine	79	4.6 (−12.1 to 24.4)	0.613	0.985
Cystine	79	−7.3 (−36.0 to 34.3)	0.690	0.985
Glutamine	79	−10.4 (−22.3 to 3.3)	0.130	0.865
Glycine	79	−3.9 (−18.9 to 13.9)	0.646	0.985
Proline	79	0.0 (−13.4 to 15.5)	0.998	0.998
Tyrosine	79	−0.1 (−15.8 to 18.7)	0.995	0.998
Alanine	79	−14.6 (−40.4 to 22.4)	0.390	0.985
Asparagine	79	−7.5 (−23.6 to 12.0)	0.423	0.985
**Aspartic acid**	**79**	**−22.2 (−39.2 to −0.4)**	**0.046**	**0.865**
Glutamic acid	79	−10.6 (−21.6 to 2.1)	0.098	0.865
Serine	79	−4.4 (−22.4 to 17.7)	0.670	0.985
**Hen's egg**				
Histidine	79	1.9 (−9.1 to 14.1)	0.751	0.879
Isoleucine	79	−4.5 (−21.3 to 16.0)	0.642	0.879
Leucine	79	−6.5 (−22.3 to 12.4)	0.473	0.879
Lysine	79	−10.7 (−24.4 to 5.5)	0.184	0.879
Methionine	79	−4.1 (−21.9 to 17.7)	0.688	0.879
Phenylalanine	79	−4.2 (−19.2 to 13.5)	0.621	0.879
Threonine	79	−5.1 (−26.1 to 21.8)	0.680	0.879
Tryptophan	79	−2.8 (−16.2 to 12.7)	0.703	0.879
Valine	79	−1.1 (−16.0 to 16.6)	0.897	0.897
Arginine	79	−2.4 (−18.7 to 17.0)	0.791	0.879
Cystine	79	−13.7 (−40.8 to 25.8)	0.444	0.879
Glutamine	79	−9.5 (−21.6 to 4.5)	0.176	0.879
Glycine	79	−4.7 (−21.7 to 15.9)	0.628	0.879
Proline	79	−4.2 (−18.7 to 12.9)	0.609	0.879
Tyrosine	79	−4.0 (−18.8 to 13.6)	0.637	0.879
Alanine	79	−9.7 (−36.5 to 28.5)	0.571	0.879
Asparagine	79	−2.1 (−20.0 to 19.9)	0.839	0.883
Aspartic acid	79	−18.3 (−35.4 to 3.4)	0.093	0.879
Glutamic acid	79	−11.9 (−23.9 to 2.0)	0.091	0.879
Serine	79	−10.4 (−27.5 to 10.9)	0.313	0.879
**Wheat**				
Histidine	79	−14.1 (−31.9 to 8.5)	0.202	0.908

Isoleucine	79	−29.9 (−66.7 to 47.5)	0.349	0.908
Leucine	79	−20.6 (−63.3 to 71.8)	0.558	0.908
Lysine	79	−14.2 (−45.8 to 35.8)	0.512	0.908
Methionine	79	3.4 (−41.4 to 82.3)	0.908	0.908
Phenylalanine	79	10.9 (−37.8 to 97.8)	0.726	0.908
Threonine	79	19.1 (−50.3 to 185.2)	0.695	0.908
Tryptophan	79	−9.0 (−52.9 to 76.0)	0.780	0.908
Valine	79	−17.8 (−54.8 to 49.4)	0.519	0.908
Arginine	79	−7.1 (−50.2 to 73.1)	0.816	0.908
**Cystine**	**79**	**−47.8 (−69.2 to −11.5)**	**0.016**	**0.316**
Glutamine	79	8.6 (−18.9 to 45.4)	0.581	0.908
Glycine	79	−2.6 (−24.5 to 25.5)	0.837	0.908
Proline	79	−28.4 (−50.7 to 4.0)	0.079	0.791
Tyrosine	79	14.5 (−41.3 to 123.3)	0.691	0.908
Alanine	79	52.3 (−65.8 to 579.3)	0.581	0.908
Asparagine	79	−4.2 (−51.7 to 90.2)	0.903	0.908
Aspartic acid	79	−37.9 (−76.0 to 60.7)	0.326	0.908
Glutamic acid	79	8.9 (−11.2 to 33.5)	0.413	0.908
Serine	79	−16.1 (−49.1 to 38.2)	0.489	0.908

Adjusted percentage differences compare partial/unrestricted with complete avoidance/minimal intake. Amino acid concentrations were log-transformed, and coefficients were back-transformed as $100 \times (\exp[\beta] - 1)$. Models included all three foods and were adjusted for age, sex, BMI SDS, and fasting duration using HC3 standard errors. FDR adjustment was separately performed across the 20 amino acids for each food. BMI, body mass index; CI, confidence interval; FDR, false discovery rate; SDS, standard deviation score.

Supplementary Table S5. Adjusted associations between dietary restriction status and growth-related outcomes.

Food	Outcome	*N*	Adjusted β (95% CI)	Robust SE	*P* value	FDR *q* value
**Cow's milk**	**Height SDS**	**80**	**0.442 (0.011 to 0.874)**	**0.220**	**0.045**	**0.122**
	Weight SDS	80	0.343 (−0.016 to 0.702)	0.183	0.061	0.122
	BMI SDS	80	0.095 (−0.261 to 0.451)	0.182	0.602	0.602
	IGF-I SDS	80	0.306 (−0.100 to 0.712)	0.207	0.14	0.186
**Hen's egg**	Height SDS	80	0.238 (−0.175 to 0.651)	0.211	0.259	0.517
	Weight SDS	80	0.283 (−0.102 to 0.668)	0.196	0.150	0.517
	BMI SDS	80	0.159 (−0.203 to 0.520)	0.184	0.390	0.520
	IGF-I SDS	80	0.041 (−0.388 to 0.469)	0.219	0.853	0.853
**Wheat**	Height SDS	80	−0.429 (−1.658 to 0.800)	0.627	0.494	0.659
	**Weight SDS**	**80**	**−0.469 (−0.832 to −0.105)**	**0.186**	**0.012**	**0.046**
	BMI SDS	80	−0.206 (−1.680 to 1.269)	0.752	0.785	0.785
	IGF-I SDS	80	−1.042 (−2.679 to 0.596)	0.836	0.213	0.425

Adjusted β coefficients compare partial/unrestricted with complete avoidance/minimal intake. Models included all three foods. Height, weight, and BMI SDS models were adjusted for age and sex; the IGF-I SDS model was additionally adjusted for fasting duration. HC3 standard errors were used. FDR adjustment was separately performed across the four outcomes for each food. Positive values indicate higher outcome SDS among participants classified in the partial/unrestricted category. BMI, body mass index; CI, confidence interval; FDR, false discovery rate; IGF-I, insulin-like growth factor I; SDS, standard deviation score; SE, standard error.

Supplementary Table S6. Adjusted associations between plasma amino acid concentrations and growth-related outcomes.

Amino acid	Height SDS	Weight SDS	BMI SDS	IGF-I SDS
Histidine	−0.104 (−0.361 to 0.152) *P*=0.426; *q*=0.861	−0.146 (−0.385 to 0.093) *P*=0.231; *q*=0.396	−0.125 (−0.329 to 0.078) *P*=0.227; *q*=0.378	−0.077 (−0.282 to 0.128) *P*=0.461; *q*=0.926
Isoleucine	−0.139 (−0.382 to 0.104) *P*=0.263; *q*=0.861	**−0.231 (−0.443 to −0.020)†** *P*=0.032; *q*=0.191	**−0.205 (−0.390 to −0.020)†** *P*=0.030; *q*=0.263	−0.102 (−0.335 to 0.130) *P*=0.389; *q*=0.926
Leucine	−0.117 (−0.366 to 0.132) *P*=0.358; *q*=0.861	−0.201 (−0.415 to 0.014) *P*=0.067; *q*=0.191	−0.176 (−0.356 to 0.005) *P*=0.057; *q*=0.276	−0.068 (−0.286 to 0.150) *P*=0.539; *q*=0.926
Lysine	−0.176 (−0.405 to 0.054) *P*=0.133; *q*=0.838	−0.203 (−0.421 to 0.014) *P*=0.067; *q*=0.191	−0.131 (−0.301 to 0.039) *P*=0.130; *q*=0.291	−0.067 (−0.258 to 0.123) *P*=0.490; *q*=0.926
Methionine	−0.030 (−0.273 to 0.213) *P*=0.809; *q*=0.884	−0.139 (−0.353 to 0.074) *P*=0.200; *q*=0.396	−0.170 (−0.366 to 0.025) *P*=0.088; *q*=0.276	0.009 (−0.195 to 0.213) *P*=0.930; *q*=0.954
Phenylalanine	−0.087 (−0.312 to 0.138) *P*=0.449; *q*=0.861	−0.158 (−0.368 to 0.052) *P*=0.140; *q*=0.311	−0.145 (−0.315 to 0.024) *P*=0.092; *q*=0.276	−0.027 (−0.225 to 0.171) *P*=0.787; *q*=0.926
Threonine	−0.068 (−0.332 to 0.195) *P*=0.611; *q*=0.873	−0.127 (−0.358 to 0.104) *P*=0.281; *q*=0.411	−0.116 (−0.314 to 0.082) *P*=0.250; *q*=0.384	−0.072 (−0.286 to 0.141) *P*=0.507; *q*=0.926
Tryptophan	−0.063 (−0.268 to 0.141) *P*=0.544; *q*=0.873	**−0.186 (−0.361 to −0.011)†** *P*=0.037; *q*=0.191	**−0.207 (−0.404 to −0.010)†** *P*=0.039; *q*=0.263	−0.041 (−0.283 to 0.202) *P*=0.742; *q*=0.926
Valine	−0.170 (−0.412 to 0.072) *P*=0.168; *q*=0.838	−0.200 (−0.412 to 0.011) *P*=0.063; *q*=0.191	−0.133 (−0.317 to 0.051) *P*=0.156; *q*=0.313	−0.058 (−0.279 to 0.163) *P*=0.606; *q*=0.926
Arginine	−0.091 (−0.339 to 0.158) *P*=0.474; *q*=0.861	−0.172 (−0.379 to 0.035) *P*=0.103; *q*=0.257	−0.158 (−0.345 to 0.028) *P*=0.097; *q*=0.276	0.054 (−0.150 to 0.257) *P*=0.605; *q*=0.926
Cystine	−0.129 (−0.335 to 0.077) *P*=0.219; *q*=0.861	**−0.196 (−0.358 to −0.033)†** *P*=0.018; *q*=0.191	**−0.162 (−0.306 to −0.018)†** *P*=0.028; *q*=0.263	0.023 (−0.196 to 0.243) *P*=0.835; *q*=0.927
Glutamine	0.037 (−0.188 to 0.262) *P*=0.747; *q*=0.884	−0.067 (−0.266 to 0.132) *P*=0.508; *q*=0.607	−0.110 (−0.273 to 0.053) *P*=0.186; *q*=0.339	0.116 (−0.120 to 0.351) *P*=0.336; *q*=0.926

Glycine	−0.122 (−0.357 to 0.113) *P*=0.309; *q*=0.861	−0.130 (−0.347 to 0.086) *P*=0.237; *q*=0.396	−0.058 (−0.257 to 0.140) *P*=0.565; *q*=0.627	0.039 (−0.138 to 0.215) *P*=0.668; *q*=0.926
Proline	0.011 (−0.245 to 0.267) *P*=0.935; *q*=0.935	−0.066 (−0.296 to 0.165) *P*=0.576; *q*=0.607	−0.105 (−0.325 to 0.116) *P*=0.352; *q*=0.463	0.055 (−0.154 to 0.265) *P*=0.604; *q*=0.926
Tyrosine	−0.035 (−0.290 to 0.220) *P*=0.789; *q*=0.884	−0.123 (−0.351 to 0.104) *P*=0.288; *q*=0.411	−0.147 (−0.337 to 0.044) *P*=0.131; *q*=0.291	0.006 (−0.193 to 0.204) *P*=0.954; *q*=0.954
Alanine	0.028 (−0.239 to 0.294) *P*=0.840; *q*=0.884	−0.004 (−0.230 to 0.221) *P*=0.970; *q*=0.970	−0.029 (−0.247 to 0.190) *P*=0.798; *q*=0.798	−0.051 (−0.333 to 0.232) *P*=0.726; *q*=0.926
Asparagine	−0.049 (−0.286 to 0.188) *P*=0.685; *q*=0.884	−0.097 (−0.327 to 0.133) *P*=0.409; *q*=0.545	−0.083 (−0.265 to 0.099) *P*=0.370; *q*=0.463	0.038 (−0.150 to 0.226) *P*=0.694; *q*=0.926
Aspartic acid	−0.146 (−0.351 to 0.059) *P*=0.162; *q*=0.838	−0.054 (−0.240 to 0.133) *P*=0.574; *q*=0.607	0.064 (−0.112 to 0.240) *P*=0.477; *q*=0.562	−0.047 (−0.215 to 0.121) *P*=0.582; *q*=0.926
Glutamic acid	0.065 (−0.174 to 0.304) *P*=0.593; *q*=0.873	0.070 (−0.163 to 0.304) *P*=0.556; *q*=0.607	0.057 (−0.174 to 0.288) *P*=0.631; *q*=0.664	0.042 (−0.202 to 0.286) *P*=0.734; *q*=0.926
Serine	−0.178 (−0.391 to 0.035) *P*=0.102; *q*=0.838	**−0.197 (−0.394 to −0.001)†** *P*=0.049; *q*=0.191	−0.105 (−0.294 to 0.083) *P*=0.273; *q*=0.390	−0.030 (−0.203 to 0.142) *P*=0.730; *q*=0.926

Values are adjusted β coefficients (95% CI) per 1-SD increase in log-transformed amino acid concentration, with nominal *P* and FDR-adjusted q values. All analyses included 79 participants. Models were adjusted for age, sex, and fasting duration; IGF-I SDS models were additionally adjusted for BMI SDS. HC3 standard errors were used. FDR adjustment was separately performed across the 20 amino acids for each outcome. †Nominal $P < 0.05$; no associations remained significant after FDR correction. BMI, body mass index; CI, confidence interval; FDR, false discovery rate; IGF-I, insulin-like growth factor I; SDS, standard deviation score.

Supplementary Table S7. Sensitivity analysis excluding wheat dietary restriction status from the model.

Amino acid	*N*	Adjusted difference, % (95% CI)	*P* value	FDR *q* value
**Cow's milk**				
Histidine	79	2.6 (−7.7 to 14.1)	0.629	0.986
Isoleucine	79	−0.1 (−16.6 to 19.7)	0.991	0.991
Leucine	79	1.5 (−14.3 to 20.1)	0.864	0.991
Lysine	79	−0.6 (−14.6 to 15.7)	0.935	0.991
Methionine	79	4.6 (−13.9 to 27.0)	0.651	0.986
Phenylalanine	79	4.5 (−11.2 to 23.1)	0.593	0.986
Threonine	79	−14.7 (−31.5 to 6.1)	0.154	0.769
Tryptophan	79	−3.2 (−16.7 to 12.4)	0.667	0.986
Valine	79	2.7 (−12.2 to 20.1)	0.740	0.986
Arginine	79	4.7 (−11.4 to 23.6)	0.593	0.986
Cystine	79	−6.7 (−35.8 to 35.6)	0.717	0.986
Glutamine	79	−10.5 (−22.2 to 3.1)	0.124	0.769
Glycine	79	−3.9 (−18.8 to 13.8)	0.646	0.986
Proline	79	0.3 (−13.4 to 16.3)	0.964	0.991
Tyrosine	79	−0.2 (−15.5 to 17.8)	0.982	0.991
Alanine	79	−15.0 (−39.5 to 19.5)	0.351	0.986
Asparagine	79	−7.5 (−22.9 to 11.1)	0.406	0.986
**Aspartic acid**	**79**	**−21.8 (−38.8 to −0.1)**	**0.049**	**0.769**
Glutamic acid	79	−10.6 (−21.6 to 1.9)	0.094	0.769
Serine	79	−4.3 (−22.0 to 17.5)	0.677	0.986
**Hen's egg**				
Histidine	79	2.1 (−9.0 to 14.5)	0.726	0.854
Isoleucine	79	−4.0 (−21.2 to 16.9)	0.683	0.854
Leucine	79	−6.2 (−22.1 to 12.8)	0.496	0.854
Lysine	79	−10.5 (−24.1 to 5.5)	0.187	0.854
Methionine	79	−4.2 (−21.7 to 17.4)	0.681	0.854
Phenylalanine	79	−4.3 (−19.1 to 13.1)	0.604	0.854
Threonine	79	−5.4 (−26.4 to 21.6)	0.667	0.854
Tryptophan	79	−2.7 (−16.0 to 12.7)	0.714	0.854
Valine	79	−0.8 (−15.9 to 17.0)	0.923	0.923
Arginine	79	−2.3 (−18.4 to 17.0)	0.798	0.887
Cystine	79	−12.9 (−40.5 to 27.5)	0.477	0.854
Glutamine	79	−9.6 (−21.6 to 4.3)	0.168	0.854
Glycine	79	−4.7 (−21.6 to 15.8)	0.629	0.854
Proline	79	−3.8 (−18.2 to 13.2)	0.645	0.854
Tyrosine	79	−4.1 (−18.7 to 13.0)	0.615	0.854
Alanine	79	−10.2 (−37.1 to 28.1)	0.552	0.854
Asparagine	79	−2.0 (−19.9 to 19.8)	0.842	0.887
Aspartic acid	79	−17.8 (−34.7 to 3.6)	0.097	0.854
Glutamic acid	79	−12.0 (−24.0 to 1.9)	0.088	0.854
Serine	79	−10.2 (−27.4 to 11.2)	0.325	0.854

Adjusted percentage differences compare partial/unrestricted with complete avoidance/minimal intake. Amino acid concentrations were log-transformed, and coefficients were back-transformed as 100 × (exp[β] − 1). Models included hen's egg and cow's milk dietary restriction status variables and were adjusted for age, sex, BMI SDS, and fasting duration using HC3 standard errors. FDR adjustment was separately performed across the 20 amino acids for each food. BMI, body mass index; CI, confidence interval; FDR, false discovery rate; SDS, standard deviation score.

Supplementary Table S8. Ordinal three-category sensitivity analysis.

Amino acid	*N*	Adjusted difference, % (95% CI)	*P* value	FDR *q* value
**Cow's milk**				
Histidine	79	1.8 (−3.9 to 7.7)	0.546	0.983
Isoleucine	79	−0.9 (−10.2 to 9.5)	0.865	0.983
Leucine	79	0.4 (−8.5 to 10.1)	0.939	0.983
Lysine	79	−0.9 (−8.7 to 7.6)	0.835	0.983
Methionine	79	1.4 (−8.8 to 12.6)	0.802	0.983
Phenylalanine	79	2.0 (−6.9 to 11.8)	0.668	0.983
Threonine	79	−7.2 (−17.8 to 4.8)	0.229	0.983
Tryptophan	79	−2.7 (−10.5 to 5.7)	0.520	0.983
Valine	79	1.2 (−7.1 to 10.3)	0.779	0.983
Arginine	79	2.1 (−7.1 to 12.2)	0.663	0.983
Cystine	79	−4.8 (−21.8 to 15.8)	0.621	0.983
Glutamine	79	−6.1 (−12.9 to 1.3)	0.102	0.930
Glycine	79	−2.3 (−10.9 to 7.1)	0.619	0.983
Proline	79	−0.2 (−7.8 to 8.0)	0.957	0.983
Tyrosine	79	−0.1 (−8.7 to 9.3)	0.983	0.983
Alanine	79	−7.2 (−23.3 to 12.2)	0.439	0.983
Asparagine	79	−5.2 (−14.1 to 4.6)	0.287	0.983
**Aspartic acid**	**79**	**−15.6 (−25.5 to −4.3)**	**0.008**	**0.160**
Glutamic acid	79	−5.3 (−11.9 to 1.8)	0.139	0.930
Serine	79	−3.6 (−13.8 to 7.9)	0.526	0.983
**Hen's egg**				
Histidine	79	0.1 (−5.8 to 6.4)	0.965	0.965
Isoleucine	79	−5.6 (−15.5 to 5.6)	0.315	0.525
Leucine	79	−6.5 (−15.8 to 3.8)	0.206	0.516
Lysine	79	−7.5 (−15.4 to 1.2)	0.088	0.433
Methionine	79	−6.6 (−17.2 to 5.4)	0.267	0.525
Phenylalanine	79	−2.9 (−12.4 to 7.6)	0.570	0.600
Threonine	79	−6.7 (−18.3 to 6.4)	0.300	0.525
Tryptophan	79	−2.9 (−11.2 to 6.1)	0.508	0.600
Valine	79	−3.5 (−12.1 to 5.9)	0.448	0.600
Arginine	79	−3.2 (−13.0 to 7.6)	0.544	0.600
Cystine	79	−15.4 (−31.9 to 5.1)	0.130	0.433
Glutamine	79	−6.6 (−14.5 to 2.0)	0.129	0.433
Glycine	79	−5.8 (−15.8 to 5.3)	0.293	0.525
Proline	79	−3.8 (−11.4 to 4.5)	0.357	0.549
Tyrosine	79	−3.7 (−12.8 to 6.5)	0.465	0.600
Alanine	79	−6.3 (−23.2 to 14.4)	0.524	0.600
Asparagine	79	−7.7 (−17.6 to 3.3)	0.164	0.468
**Aspartic acid**	**79**	**−18.1 (−28.0 to −6.8)**	**0.002**	**0.049**
Glutamic acid	79	−7.8 (−15.1 to 0.2)	0.055	0.433
Serine	79	−10.4 (−20.7 to 1.2)	0.076	0.433
**Wheat**				
Histidine	79	−7.5 (−16.8 to 2.8)	0.146	0.593
Isoleucine	79	−16.3 (−33.4 to 5.3)	0.129	0.593

Leucine	79	−12.1 (−29.8 to 10.1)	0.262	0.659
Lysine	79	−7.9 (−20.3 to 6.4)	0.264	0.659
Methionine	79	−7.0 (−24.1 to 13.8)	0.480	0.730
Phenylalanine	79	1.9 (−14.7 to 21.7)	0.837	0.893
Threonine	79	−1.9 (−25.8 to 29.7)	0.893	0.893
Tryptophan	79	−9.7 (−26.7 to 11.3)	0.339	0.667
Valine	79	−11.9 (−26.6 to 5.8)	0.175	0.593
Arginine	79	−4.9 (−21.6 to 15.5)	0.615	0.73
**Cystine**	**79**	**−31.7 (−51.5 to −3.9)**	**0.029**	**0.576**
Glutamine	79	2.7 (−7.5 to 14.0)	0.621	0.730
Glycine	79	−4.2 (−15.9 to 9.1)	0.519	0.730
Proline	79	−7.6 (−22.4 to 9.9)	0.371	0.667
Tyrosine	79	1.9 (−17.3 to 25.5)	0.862	0.893
Alanine	79	13.9 (−28.1 to 80.3)	0.580	0.730
Asparagine	79	−8.7 (−26.1 to 12.8)	0.400	0.667
Aspartic acid	79	−22.0 (−41.9 to 4.6)	0.097	0.593
Glutamic acid	79	4.5 (−4.5 to 14.3)	0.341	0.667
Serine	79	−11.6 (−26.2 to 5.8)	0.178	0.593

Dietary restriction status was coded as 0, complete avoidance/minimal intake; 1, partial intake; and 2, unrestricted intake. Estimates are adjusted percentage differences per one-category shift toward less restriction. Amino acid concentrations were log-transformed, and coefficients were back-transformed as $100 \times (\exp[\beta] - 1)$. Models included all three ordinal dietary restriction status variables and were adjusted for age, sex, BMI SDS, and fasting duration using HC3 standard errors. FDR adjustment was separately performed across the 20 amino acids for each food. The model assumes linear, equally spaced category effects. BMI, body mass index; CI, confidence interval; FDR, false discovery rate; SDS, standard deviation score.

Supplementary Table S9. Food-specific allergy-restricted analyses.

Amino acid	*N*	Adjusted difference, % (95% CI)	*P* value	FDR *q* value
**Cow's milk allergy only**				
Histidine	42	−2.5 (−15.4 to 12.4)	0.729	0.810
Isoleucine	42	−14.2 (−35.2 to 13.7)	0.287	0.485
Leucine	42	−13.7 (−34.4 to 13.5)	0.291	0.485
Lysine	42	−17.6 (−34.2 to 3.3)	0.093	0.311
Methionine	42	−8.3 (−37.0 to 33.4)	0.650	0.804
Phenylalanine	42	1.6 (−26.0 to 39.4)	0.923	0.923
**Threonine**	**42**	**−34.2 (−46.6 to −18.9)**	**<0.001**	**0.002**
Tryptophan	42	−13.5 (−32.8 to 11.4)	0.262	0.485
Valine	42	−10.4 (−29.6 to 14.1)	0.374	0.576
Arginine	42	−2.3 (−29.5 to 35.3)	0.888	0.923
**Cystine**	**42**	**−50.1 (−70.4 to −15.8)**	**0.009**	0.092
Glutamine	42	−13.0 (−31.9 to 11.2)	0.266	0.485
Glycine	42	−16.3 (−36.9 to 10.9)	0.216	0.485
Proline	42	−3.5 (−18.1 to 13.8)	0.674	0.804
Tyrosine	42	−5.6 (−28.5 to 24.6)	0.683	0.804
Alanine	42	−29.9 (−50.7 to −0.3)	0.048	0.311
Asparagine	42	−12.6 (−40.4 to 28.1)	0.489	0.699
Aspartic acid	42	−29.9 (−51.7 to 1.9)	0.063	0.311
Glutamic acid	42	−12.7 (−25.1 to 1.6)	0.080	0.311
Serine	42	−16.7 (−38.8 to 13.4)	0.245	0.485
**Hen's egg allergy only**				
Histidine	56	5.9 (−7.4 to 21.0)	0.405	0.94
Isoleucine	56	3.4 (−18.0 to 30.4)	0.778	0.94
Leucine	56	2.3 (−17.3 to 26.5)	0.836	0.94
Lysine	56	−5.3 (−21.4 to 14.0)	0.564	0.94
Methionine	56	9.2 (−13.8 to 38.4)	0.466	0.94
Phenylalanine	56	1.9 (−15.9 to 23.5)	0.845	0.94
Threonine	56	9.7 (−16.4 to 44.0)	0.503	0.94
Tryptophan	56	1.0 (−14.7 to 19.5)	0.909	0.94
Valine	56	7.9 (−11.0 to 30.8)	0.442	0.94
Arginine	56	4.6 (−15.0 to 28.8)	0.671	0.94
Cystine	56	−2.3 (−38.6 to 55.6)	0.923	0.94
Glutamine	56	−1.5 (−17.6 to 17.7)	0.866	0.94
Glycine	56	3.7 (−17.4 to 30.3)	0.755	0.94
Proline	56	0.7 (−16.6 to 21.7)	0.940	0.94
Tyrosine	56	4.2 (−14.2 to 26.6)	0.679	0.94
Alanine	56	−2.1 (−34.3 to 45.9)	0.917	0.94
Asparagine	56	11.1 (−10.9 to 38.6)	0.350	0.94
Aspartic acid	56	−12.4 (−34.0 to 16.3)	0.360	0.94
Glutamic acid	56	−5.7 (−20.6 to 12.1)	0.508	0.94
Serine	56	−3.3 (−24.5 to 24.0)	0.793	0.94
**Wheat allergy only**				
Histidine	14	−0.6 (−38.2 to 59.8)	0.980	0.987
Isoleucine	14	−9.9 (−62.0 to 113.9)	0.813	0.987

Leucine	14	5.1 (−60.4 to 179.1)	0.921	0.987
Lysine	14	0.5 (−43.6 to 78.9)	0.987	0.987
Methionine	14	39.7 (−50.8 to 296.4)	0.530	0.987
Phenylalanine	14	35.9 (−40.3 to 209.5)	0.465	0.987
Threonine	14	52.7 (−60.1 to 483.9)	0.536	0.987
Tryptophan	14	23.3 (−41.5 to 159.9)	0.582	0.987
Valine	14	7.7 (−48.1 to 123.6)	0.842	0.987
Arginine	14	18.4 (−48.5 to 172.0)	0.691	0.987
Cystine	14	−46.3 (−95.8 to 586.5)	0.633	0.987
Glutamine	14	41.8 (−22.0 to 157.8)	0.252	0.987
Glycine	14	12.7 (−48.0 to 144.2)	0.762	0.987
**Proline**	**14**	**−32.6 (−50.6 to −8.0)**	**0.013**	**0.130**
Tyrosine	14	65.0 (−40.8 to 359.7)	0.338	0.987
Alanine	14	72.2 (−86.7 to 2124.7)	0.677	0.987
**Asparagine**	**14**	**41.1 (11.1 to 79.2)**	**0.005**	**0.094**
Aspartic acid	14	−40.1 (−95.7 to 735.4)	0.703	0.987
Glutamic acid	14	30.3 (−33.4 to 155.0)	0.440	0.987
Serine	14	−3.9 (−50.0 to 84.9)	0.905	0.987

Analyses were restricted to participants with the corresponding food allergy. Adjusted percentage differences compare partial/unrestricted with complete avoidance/minimal intake within each subgroup. Amino acid concentrations were log-transformed, and coefficients were back-transformed as 100 × (exp[β] − 1). Models were adjusted for age, sex, BMI SDS, and fasting duration using HC3 standard errors. FDR adjustment was separately performed across the 20 amino acids within each subgroup. BMI, body mass index; CI, confidence interval; FDR, false discovery rate; SDS, standard deviation score.

Supplementary Table S10. Multivariate associations with the overall plasma amino acid profile.

**Predictor**	**Partial R^2**	***P* value**	***q* value**
Hen's egg dietary restriction status	0.0103	0.486	0.506
Cow's milk dietary restriction status	0.0099	0.506	0.506
Wheat dietary restriction status	0.0107	0.465	0.506
Height SDS	0.0124	0.375	0.500
Weight SDS	0.0318	0.040	0.130
BMI SDS	0.0285	0.065	0.130
IGF-I SDS	0.0039	0.941	0.941

Analyses used standardized log-transformed concentrations of 20 plasma amino acids and 4,999 permutations. Models of dietary restriction status included all three foods and were adjusted for age, sex, BMI SDS, and fasting duration. Growth-related models used the covariate structures described in the Methods section. q values were calculated within each analysis family. PERMDISP showed no evidence of unequal dispersion between dietary restriction groups (all $P \geq 0.751$). BMI, body mass index; IGF-I, insulin-like growth factor I; SDS, standard deviation score.

Supplementary Figures

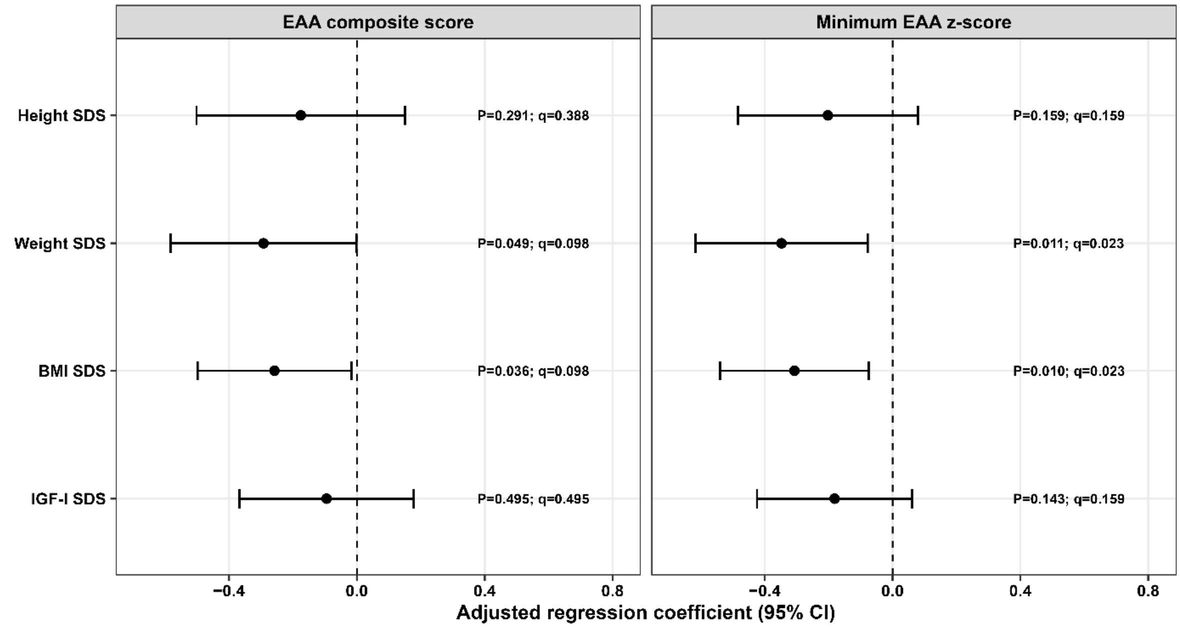

Supplementary Figure S1. Adjusted associations between essential amino acid summary measures and growth-related outcomes

The dots and horizontal lines indicate the adjusted regression coefficients and 95% confidence intervals, respectively. The EAA composite score was calculated as the mean of the standardized log concentrations of the nine EAAs, and the minimum EAA z-score was defined as the lowest of the nine standardized log concentrations for each participant. The models were adjusted for age, sex, and fasting duration; the IGF-I SDS model was additionally adjusted for BMI SDS. *P* and Benjamini–Hochberg-adjusted q values are shown in the figure (N = 79). BMI, body mass index; EAA, essential amino acid; IGF-I, insulin-like growth factor I; SDS, standard deviation score.

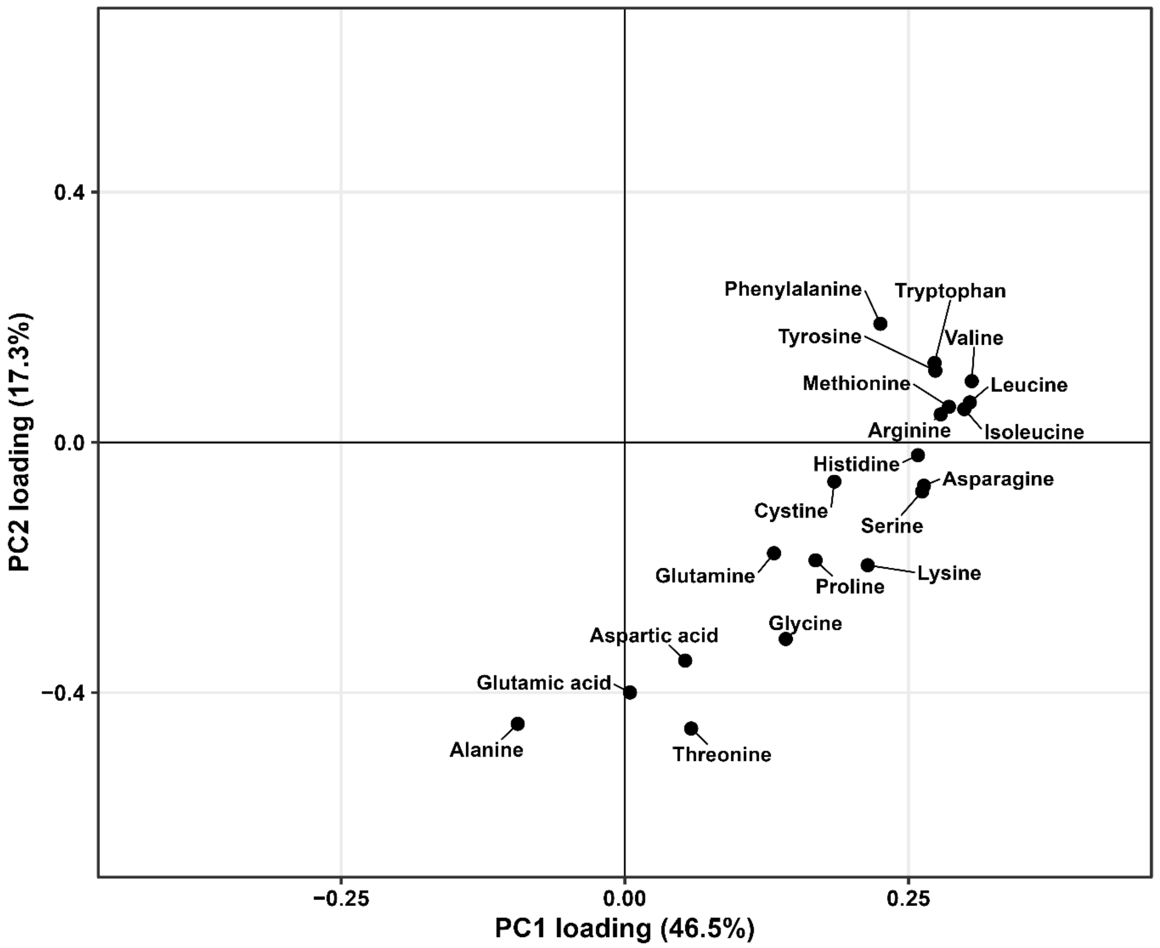

Supplementary Figure S2. Principal component loading plot of the 20-amino-acid profile

Loadings of the 20 standardized log-transformed plasma amino acid concentrations on PC1 and PC2 are shown. PC1 and PC2 explained 46.5% and 17.3% of total variance, respectively. Amino acids positioned in similar directions tended to vary, and the distance from the origin indicated the magnitude of their contribution. PC, principal component.
